# Mortality among patients with tuberculosis requiring intensive care: a retrospective cohort study

**DOI:** 10.1186/1471-2334-10-54

**Published:** 2010-03-07

**Authors:** Denise R Silva, Diego M Menegotto, Luis F Schulz, Marcelo B Gazzana, Paulo TR Dalcin

**Affiliations:** 1 Faculdade de Medicina, Universidade Federal do Rio Grande do Sul (UFRGS); Hospital de Clínicas de Porto Alegre, RS, Brazil

## Abstract

**Background:**

To describe the characteristics of patients with tuberculosis (TB) requiring intensive care and to identify the factors that predicts in-hospital mortality in a city of a developing country with intermediate-to-high TB endemicity.

**Methods:**

We conducted a retrospective, cohort study, between November 2005 and November 2007. The patients with TB requiring intensive care were included. Predictors of mortality were assessed. The primary outcome was the in-hospital mortality.

**Results:**

During the study period, 67 patients with TB required intensive care. Of them, 62 (92.5%) had acute respiratory failure and required mechanical ventilation. Forty-four (65.7%) patients died. Coinfection with human immunodeficiency virus was present in 46 (68.7%) patients. Early intensive care unit admission and ventilator-associated pneumonia were independently associated with the in-hospital mortality.

**Conclusions:**

In this study we found a high mortality rate in TB patients requiring intensive care, especially in those with an early ICU admission.

## Background

Across the world tuberculosis (TB) remains an important public health problem, especially in developing countries. One third of the world's population is infected with *Mycobacterium tuberculosis*. Brazil is ranking 15^th ^among the 22 high-burden countries that collectively account for 80% of TB cases globally. The incidence of TB was of 50 cases/100,000 population/yr in 2006, and recently reached approximately 100 cases/100,000 population in the city of Porto Alegre (southern Brazil) [1]. Every year, almost 2 million people die of TB, most of them in low- and middle-income countries. The annual death rate from TB in Brazil was estimated at 4.0/100,000 population/yr in 2006 [2].

Despite the availability of curative therapy, a large proportion of patients with TB are being hospitalized. In-hospital mortality rates remain high, particularly among patients with TB requiring intensive care unit (ICU) admission. These cases represent 1-3% of all patients with TB [3]. Acute respiratory failure (ARF) caused by TB necessitating mechanical ventilation (MV) has been associated with mortality rates between 25.9% and 81% [3-6]. Furthermore, such patients have a prognosis significantly worse than patients with nontuberculous pneumonia requiring MV [6].

Some studies reported a few factors that contribute to mortality among critically ill patients with TB. Disseminated disease, usually in the setting of human immunodeficiency virus (HIV) infection, has been recognized as an important predictor of death. Other factors that can influence mortality rates are extensive fibrocavitary disease and consolidations on chest radiographs. Acute respiratory distress syndrome (ARDS), sepsis and multiple organ failure (MOF) also carry a very high mortality [5-7].

The purpose of this study was to describe the characteristics of patients with TB requiring intensive care, and to identify the factors that predict in-hospital mortality in a city of a developing country with intermediate-to-high TB endemicity.

## Methods

This study was conducted in Porto Alegre, Brazil, from November 2005 through November 2007. Adult patients with TB that were admitted to the ICU of the Clinics Hospital of Porto Alegre (HCPA) were identified retrospectively. The HCPA is a general, tertiary care, university-affiliated hospital with 750 beds (34 adult ICU beds) and is a reference center for HIV in southern Brazil. Porto Alegre is the city with the highest acquired immunodeficiency syndrome (AIDS) incidence in Brazil (68.7 cases/100.000 population) [1]. In our hospital, in 2008 for example, we had approximately 29.000 hospitalizations per year, with 185 cases of pulmonary TB. In addition, the number of outpatient visits was 551.968, but we do not have data on TB treatment, because our hospital does not provide TB treatment for outpatients; in Brazil, patients are treated in public outpatient health care services. The medical records of patients were reviewed, and predictors of mortality were assessed. The protocol was submitted to the Ethics Committee at HCPA and an approval was obtained. A waiver of consent was approved and the investigators assigned a confidentiality term.

Pulmonary TB is diagnosed according to the following criteria established in the Brazilian Guidelines for Tuberculosis [8]: 1) detection by a direct test (Ziehl-Neelsen [ZN] method) - two positive samples; or 2) detection by a direct test (ZN method) - one positive sample and a positive culture result for *Mycobacterium tuberculosis *(in Löwenstein-Jensen [LJ] medium); or 3) detection by a direct test (ZN method) - one positive sample and radiological findings compatible with TB; or 4) only a positive culture result for *Mycobacterium tuberculosis *(in LJ medium); or 5) presence of clinical, epidemiologic and radiographic findings compatible with TB. The diagnosis of extra-pulmonary TB was based on clinical and/or complementary tests according with the location.

The following data were collected in a standardized questionnaire: demographic data (sex, age, race, years of schooling), smoking status, alcoholism, injection drug use, clinical form of TB, symptoms at admission, methods of diagnostic, presence of comorbidities, prior TB treatment, drug regimen, interval from hospital admission until initiation of treatment, delayed treatment (failure to initiate treatment within the first 24 hours after admission to hospital), reasons for ICU admission, interval from hospital admission until ICU admission (early ICU [admitted directly or transferred to an ICU within 4 days of admission] and late ICU [admitted to an ICU after 4 days of hospitalization]), ARF, ventilator-associated pneumonia (VAP), Acute Physiologic and Chronic Health Evaluation (APACHE) II scores, presence of organ failures, ARDS, sepsis, septic shock, length of hospital and ICU stay, length of mechanical ventilation, laboratory investigations (white cell count, hemoglobin, coagulation profile, liver and renal function parameters, albumin, C-reactive protein, blood gas analysis), Glasgow coma score, urine output, use of vasoactive drugs, hospitalization outcome (death or discharge), and outcome after discharge (cure, dropout, death). Data after discharge were obtained from SINAN (National System of Information on Notifiable Diseases). SINAN is a database from the Brazilian government which stores information concerning all notifiable infectious and contagious diseases. The duration of follow-up period was one year.

A diagnosis of respiratory failure was made after the determination of arterial oxygen pressure (PaO_2_) level of less than 60 mmHg or arterial oxygen saturation (SaO_2_) < 90%, with or without elevation of arterial PCO_2_. ARDS was diagnosed based on the criteria defined in the American-European consensus conference [9]. For the diagnosis of organ failure, the criteria by Knaus [10] were used. Multiple organ failure (MOF) was defined as the failure of more than one organ. Ventilator-associated pneumonia (VAP) was diagnosed based on the American Thoracic Society (ATS) criteria [11]. Anemia was defined by hemoglobin levels < 13.5 g/dL for males and < 12.0 g/dL for females. Hypoalbuminemia was considered when serum albumin levels were less than 3.5 g/dL.

Data analysis was performed using SPSS 14.0 (Statistical Package for the Social Sciences, Chicago, Illinois). Data were presented as number of cases, mean ± standard deviation (SD), or median with interquartile range. Categorical comparisons were performed by chi-square test using Yates's correction if indicated or by Fisher's exact test. Continuous variables were compared using the *t*-test or Wilcoxon test. Variables with p < 0.20 in the univariate analysis were analyzed in the multivariate analyses. We have chosen this widespread adopted cutoff because using a more traditional level (*P *< 0.05) might fail to identify variables known to be important. In addition, confusion variables can affect estimates even when their level of significance does not reach 0.05. Kaplan-Meier survival curves were used to analyze the survival of patients. Those factors significantly associated with survival were further analyzed with the Cox proportional hazard model (method forward) adjusted by sex and age. Hazard ratio (HR) with 95% confidence interval (CI) was used to report the results. A two-sided p value < 0.05 was considered significant for all analyses.

## Results

Between November 2005 and November 2007, 67 patients with TB required intensive care in our hospital. Of them, 62 (92.5%) had ARF and required MV. The mean age of all patients was 43.2 ± 14.1 years and males slightly outnumbered female patients (55.2% vs. 44.8%). A history of previous TB was present in only 6 patients (9%). Comorbid illnesses were identified in 58 (86.6%) patients, with coinfection with HIV being the most common, present in 46 (68.7%) patients. The median CD4/mm^3 ^count of these patients was 83 (range: 34 - 145), and only 6 (13.0%) were receiving highly active anti-retroviral therapy (HAART) at the time of hospitalization. A history of alcoholism was present in 25 (37.3%) patients and 20 (30%) were current smokers. Demographic and clinical characteristics did not differ between the survivors and those that died (Table 1).

**Table 1:** Demographic and clinical characteristics of hospitalized patients with TB requiring intensive care

	Nonsurvivors (n = 44)	Survivors (n = 23)	p-value
Age, yr	44.2 ± 13.8	41.2 ± 14.8	0.414
Male sex	20 (45.5)	10 (43.5)	0.999
White race	34 (77.3)	14 (60.9)	0.259
< 8 yr of schooling	32 (82.1)	18 (85.7)	0.999
Current smoker	11 (26.8)	9 (45.0)	0.259
Presence of any comorbidity	39 (88.6)	19 (82.6)	0.481
HIV	30 (71.4)	16 (72.7)	0.999
CD4 count	91 (83-229)	45.5 (25.5-116.8)	0.079
Alcoholism	13 (29.5)	12 (52.2)	0.121
Drug use	7 (15.9)	6 (26.1)	0.344
Diabetes	5 (11.4)	1 (4.3)	0.656
Corticosteroid use	6 (13.6)	0	0.087
Transplantation	3 (6.8)	0	0.546
Renal chronic disease	2 (4.5)	1 (4.3)	0.999
Hepatic disease	1 (2.3)	0	0.999
Desnutrition	1 (2.3)	3 (13.0)	0.113
Neoplasia	2 (4.5)	0	0.542

Data are presented as mean ± SD, n (%), or median (interquartile range). HIV: human immunodeficiency virus.

Twenty four (35.8%) patients had pulmonary disease only, 21 (31.3%) had extrapulmonary disease only, and 22 (32.8%) had association of pulmonary and extrapulmonary disease. The most common symptoms were fever (44.8%), loss of weight (38.8%), cough (29.9%), and neurologic ones (22.4%). Reticular infiltrate (35.8%) was the most common radiographic finding, followed by consolidation (23.9%). Forty six patients collected a sputum sample, of whom 25 (54.3%) were sputum-smear positive. Mycobacterial cultures were positive in 35 (52.2%) patients (13 bronchoalveolar lavage, 8 pleural effusion, 3 spontaneous sputum, 3 bone marrow, 2 cerebrospinal fluid, 2 blood, 1 induced sputum, 1 fine needle aspiration of lymphonode, 1 ascitic fluid, 1 urine). In 9 (13.4%), TB diagnosis was based on clinical, epidemiologic and radiographic findings. The median interval from hospital admission until initiation of treatment was 3 days (range: 1 - 8).

The primary cause for ICU admission was ARF in 42 (62.7%) patients. Other reasons were cardiopulmonary arrest (10.4%), septic shock (7.5%), sepsis (6.0%), and altered sensorium (6.0%). The mean APACHE II score was 22.8 ± 6.8. Among ICU admissions, 18.9% and 81.1% were classified as early and late, respectively. At ICU admission, most of the patients had anemia (92.5%), with more than a half (52.2%) of these having hemoglobin levels < 9 g/dL. The white blood cell (WBC) count was less than 4000 mm^-3 ^in 14 patients (20.9%) and more than 12000 mm^-3 ^in 13 patients (19.4%). Hypoalbuminemia was present in 45 (67.2%) patients. Mean serum albumin level was 2.2 ± 0.8 g/dL.

Although only 6.0% and 7.5% of patients had sepsis and septic shock, respectively, most of the patients developed sepsis (95.5%) and septic shock (83.6%) during ICU stay. Only 9 (13.4%) patients developed ARDS. During the course of ICU stay, 20 (29.9%) patients were diagnosed with VAP, and over 80% of the patients had MOF. Almost all patients (95.5%) developed acute renal failure. Out of these patients, 29.9% required dialysis. The median duration of hospitalization was 25 days (range: 12.8 - 50), and the median ICU stay was 10 days (range: 3 - 16.8).

**Figure 1:**
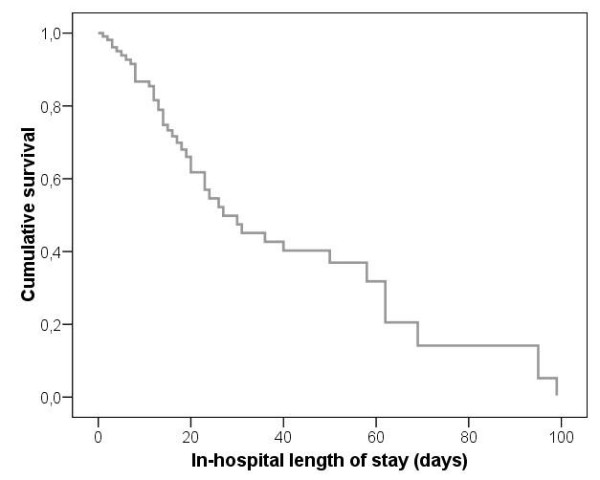
**Mortality among patients with tuberculosis requiring intensive care: survival function at mean of covariates**.

Overall, 44 (65.7%) patients died, of whom 38 (56.7%) died in ICU and 6 (8.9%) died after been transferred to the ward. The Kaplan-Meier survival curve is showed in Figure 1. Of the 23 patients who were discharged from the hospital, 17 (73.9%) were cured, 2 (8.7%) abandoned the treatment after 1 month, and 4 (17.4%) died after a mean of 3.7 ± 2.5 months. As shown in Table 2, early ICU admission was associated with mortality (p = 0.004). Early ICU admission was also associated with delayed diagnosis (p = 0.001) and acute renal failure (p = 0.030). Miliary radiologic pattern and smear-positive sputum were protective factors (p = 0.016 and 0.030, respectively). Sixty three (94%) patients used rifampicin, isoniazid and pirazynamid; 3 (4.5%) used streptomycin, isoniazid and ethambutol; 1(1.5%) used rifampicin, isoniazid and ethambutol. Only 3 patients did not receive rifampicin-based regimen because of hepatotoxicity. Nonsurvivors used more rifampicin-based regimens compared to survivors (p = 0.037). Neither hypoalbuminemia (p = 0.111) nor hepatic dysfunction (p = 0.999) had effect on mortality. We have found no association between diagnosis of HIV and mortality (p = 0.999), regardless of level of immunosuppression and use of HAART. Although not statistically significant, a higher mortality rate was found in patients with acute renal failure (p = 0.133). VAP was more common in survivors than in nonsurvivors (p = 0.041). The mean duration of mechanical ventilation was not statistically different between survivors and non-survivors (p = 0.228).

**Table 2:** Characteristics of disease presentation of hospitalized patients with TB requiring intensive care

	Nonsurvivors (n = 44)	Survivors (n = 23)	p-value
APACHE II score	22.4 ± 6.9	23.4 ± 6.8	0.625
Early ICU admission	10 (100)	0	0.004
Presence of at least one symptom	35 (81.4)	21 (91.3)	0.474
Pulmonary TB	27 (61.4)	18 (78.3)	0.261
Isolated Pulmonary TB	16 (36.4)	8 (34.8)	0.999
Isolated Extrapulmonary TB	16 (36.4)	5 (21.7)	0.343
Pulmonary + Extrapulmonary TB	12 (27.3)	10 (43.5)	0.286
Cavitary disease	3 (6.8)	2 (8.7)	0.999
Milliary radiological pattern	2 (4.5)	6 (26.1)	0.016
Smear-positive sputum	10 (38.5)	15 (75.0)	0.030
Delayed diagnosis	14 (33.3)	7 (31.8)	0.999
WBC > 12000 mm^-3^	11 (25.0)	2 (8.7)	0.192
Rifampicin-based regimens	44 (100)	20 (87.0)	0.037
Hepatotoxicity during hospitalization	5 (11.4)	6 (26.1)	0.167
Mechanical ventilation	41 (93.2)	21 (91.3)	0.999
Duration of mechanical ventilation, d	5 (2-15)	9.5 (3.75-21.3)	0.228
Sepsis	43 (97.7)	21 (93.3)	0.269
Septic shock	39 (88.6)	17 (73.9)	0.167
ARDS	5 (11.4)	4 (17.4)	0.481
Ventilator-associated pneumonia	9 (20.5)	11 (47.8)	0.041
Acute renal failure	17 (38.6)	4 (17.4)	0.133
Multiple organ failure	37 (84.1)	21 (91.3)	0.708

Data are presented as mean ± SD, n (%), or median (interquartile range). WBC: white blood cell. ARDS: acute respiratory distress syndrome.

**Figure 2:**
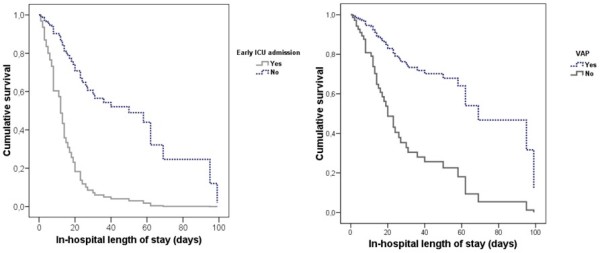
**Mortality among patients with tuberculosis requiring intensive care: survival functions for patterns of early intensive care unit (ICU) admission and for ventilator-associated pneumonia (VAP)**.

All predictors with p values ≤ 0.20 in the univariate analysis were retained in the Cox-proportional hazard model. The variables alcoholism, corticosteroid use, desnutrition, early ICU admission, miliary radiological pattern, WBC > 12000 mm^-3^, rifampicin-based regimens, hepatotoxicity, septic shock, VAP and acute renal failure, adjusted by sex and age, were introduced into the forward conditional Cox regression model, which showed that early ICU admission (HR 4.93, 95% CI 2.44 - 9.97, p < 0.001) and VAP (HR 0.26-0.12, 95% CI 0.12 - 0.59, p = 0.001) were factors affecting the in-hospital mortality rate (Figure 2).

## Discussion

In this study, we aimed to evaluate, on a retrospective basis, the risk factors associated with mortality among patients with TB requiring intensive care. Overall, we found a high in-hospital mortality rate (65.7%) in patients with TB admitted to the ICU. Using multivariate analysis, we identified early ICU admission as the most important factor independently associated with patient mortality. In addition, VAP was as a protective factor for mortality.

Our results are consistent with previous epidemiological data. Other studies have shown mortality rates up to approximately 80% in TB patients with ARF requiring MV [3-6,12]. In accordance with our findings, another study demonstrated an ICU mortality rate of 61% and an in-hospital mortality rate of 65.9% [5]. Early ICU admission was independently associated with higher mortality in multivariate analysis. This finding is consistent with a previous study [13], and may be due to the more severe disease in these patients. Actually, this appears to be a plausible explanation as they have a higher incidence of acute renal failure compared to patients with a late ICU admission. Delayed treatment was also more frequent in this group, which confirms earlier observations [13,14]. Another factor that contributes to the failure to initiate treatment within 24 hours after admission is the less classic presentation. Indeed, atypical features as sputum-smear negativity have been suggested to be associated with delays in both diagnosis and treatment and with mortality [13,15,16]. Our study showed that sputum smear-positivity and a miliary radiographic pattern were associated with a reduced risk of death. Surprisingly, we found in univariate and multivariate analysis that VAP was a further protective factor for mortality, in contrast with prior reports [4,17]. Although not statistically significant, the mean duration of mechanical ventilation was longer in survivors compared with nonsurvivors, which could explain the higher incidence of VAP in the formers.

Our study has some methodological limitations. First, the information was obtained retrospectively from chart review and probably was not as complete and accurate as when data collection is done prospectively. Certain important factors that have been suggested as risk factors for mortality could not be included. Second, it must be considered that this is a single center study with small sample size. Despite these limitations, our results provide important implications for similar demographic areas and clinical settings.

In the present study, we observed a high incidence of acute renal failure. In univariate analysis, we found that acute renal failure was associated with death. Acute renal failure is a common condition that affects many ICU patients and is a well-recognized risk factor for mortality [18,19]. However, the results reported across studies in TB patients with acute renal failure are inconsistent. Although acute renal failure was identified as an independent predictor of death in some studies [4,6,20], others [3,12] did not find a significant impact of such complication on the mortality of critically ill TB patients.

Previous studies have shown a critical course in patients with miliary TB and in HIV-infected patients with TB [6,21]. However, we did not find an association between a diagnosis of HIV and mortality in ICU patients. In fact, mortality in patients with TB-HIV coinfection seems to be related to the patient's overall degree of immunosuppression [21]. In spite of the free access to HAART in Brazil and the high level of immunosuppression of our study population, only 13% of patients were receiving HAART at the time of hospitalization, which is probably related to poor or no adherence to treatment. Even so, CD4/mm^3 ^count was not a predictor of death in these patients. Furthermore, serum albumin levels, reported by others as good predictors of CD4/mm^3 ^lymphocyte counts in patients with TB [22], were not associated with mortality in our study. The lack of association between mortality and the HIV epidemic could be explained by the fact that our non-HIV population had a high proportion of comorbidities and were also too ill with high mortality rate.

We found that patients who used rifampicin-based regimens had a higher mortality rate, even though not confirmed in multivariate analysis. One hypothesis to explain such observation is the possibility of multidrug-resistant tuberculosis (MDR-TB). However, culture and sensitivity test to antituberculosis drugs were not routinely performed, so we could not estimate the prevalence of MDR-TB in our sample. On the other hand, uncertain enteric absorption in critically ill patients [23] and subtherapeutic serum levels of rifampicin, especially in HIV-positive population [24,25], have been reported [26-28]. Drug interactions among antituberculosis drugs and HAART are also important factors to be considered. In addition, low serum albumin levels could impair drug absorption and was associated with low rifampicin and ethambutol concentrations [29]. Whereas the great majority of our study sample is composed by HIV-positive patients and hypoalbuminemia was a prevalent finding, it is possible that some patients had low serum rifampicin levels that went undetected.

To our knowledge this is the first study in Brazil that described TB cases and their outcomes in patients requiring intensive care. While effective treatment is available on an outpatient basis, TB hospitalization and mortality rates are substantially high in our center. Patients with an early ICU admission have the highest risk of death. Some factors were significant only in univariate analysis, such as acute renal failure, miliary radiological pattern, smear-positive sputum and rifampicin-based regimens. The small sample size may have lacked sufficient statistical power to reveal an association with some of the factors. These results, if confirmed in a larger prospective study, could contribute to a better understanding of characteristics associated with mortality.

## Conclusions

In conclusion, in this study we found a high mortality rate in TB patients requiring intensive care, especially in those with an early ICU admission. Nevertheless, additional studies are necessary to confirm this data, taking into account other possible risk factors for mortality like MDR-TB and low serum levels of antituberculosis drugs, potentially improving the care of critically ill TB patients.

## Competing interests

The authors declare that they have no competing interests.

## Authors' contributions

DRS participated in the conception of the study, in its design, collected the data, performed the statistical analysis and wrote the manuscript. DMM and LFS collected the data and helped to draft the manuscript. MBG participated in the conception of the study, in its design and helped to draft the manuscript. PTRD participated in the conception of the study, in its design, performed the statistical analysis and helped to draft the manuscript. All authors read and approved the final manuscript.

## Pre-publication history

The pre-publication history for this paper can be accessed here:

http://www.biomedcentral.com/1471-2334/10/54/prepub
